# Case Report: Peripheral blood T cells and inflammatory molecules in lung cancer patients with immune checkpoint inhibitor-induced thyroid dysfunction: Case studies and literature review

**DOI:** 10.3389/fonc.2022.1023545

**Published:** 2022-12-07

**Authors:** Mona A. Marie, Justin D. McCallen, Zahra S. Hamedi, Abdul Rafeh Naqash, Alexander Hoffman, Druid Atwell, Suneetha Amara, Mahvish Muzaffar, Paul R. Walker, Li V. Yang

**Affiliations:** ^1^ Department of Internal Medicine, Brody School of Medicine, East Carolina University, Greenville, NC, United States; ^2^ Department of Internal Medicine, College of Medicine, University of Oklahoma, Oklahoma City, OK, United States; ^3^ Circulogene, Birmingham, AL, United States

**Keywords:** immune checkpoint inhibitor (ICI), thyroid dysfunction and thyroiditis, lung cancer, immune-related adverse events, irAEs

## Abstract

Immunotherapy has changed the paradigm of cancer treatment, yet immune checkpoint inhibitors (ICIs) such as PD-1/PD-L1 monoclonal antibodies may cause immune-related adverse events (irAEs) in some patients. In this report, two non-small cell lung cancer (NSCLC) patients treated with nivolumab presented with checkpoint inhibitor-induced thyroid dysfunction (CITD), followed by a second irAE of pneumonitis and intestinal perforation, respectively. Increases in peripheral CD8^+^ T cells correlated with the onset of CITD in the patients. Intriguingly, common inflammatory biomarkers, including C-reactive protein (CRP) and neutrophil/lymphocyte ratio (NLR), were not consistently increased during the onset of CITD but were substantially increased during the onset of pneumonitis and intestinal perforation irAEs. The observations suggest that unlike other irAEs such as pneumonitis, CRP levels and NLR were non-contributory in diagnosing CITD, whereas T cell expansion may be associated with immunotherapy-induced thyroiditis.

## Introduction

Immune checkpoint inhibitors (ICIs) are currently the first-line treatment for multiple late-stage malignancies including non-small cell lung cancer (NSCLC). However, systemic immunostimulation by ICIs may lead to immune-related adverse events (irAEs) in some patients (1–4). Patients receiving ICIs are closely monitored for clinical symptoms and signs of irAEs as well as laboratory tests, including C reactive protein (CRP) levels, to detect occult, subclinical irAEs (1, 5).

Checkpoint inhibitor-induced thyroid dysfunction (CITD) is one of the most common irAEs, along with rash, pruritis, and diarrhea (1–3). While CITD is often detected by regular measurements of thyroid-stimulating hormone (TSH), it is unclear whether other common biomarkers also have utility in understanding the molecular mechanism and prognosis of CITD (6, 7).

Here we present two cases of patients with NSCLC treated with ICIs that developed CITD, followed by a second irAE. We describe the levels of peripheral blood biomarkers associated with CITD’s respective disease courses, including TSH, CRP, immune cell subsets, and cytokine levels. In two patients with CITD, peripheral blood CD8^+^ T cells increased in number at or preceding the time of CITD, yet the patients did not consistently demonstrate an acute change in systemic inflammatory biomarkers that were observed with the second irAEs (pneumonitis and intestinal perforation, respectively) they developed later. This study provides data that may enhance the management of CITD and prompt further investigations on the mechanisms of CITD.

## Case presentation

### Case #1

The first patient was a 55-year-old male with a five-year history of inoperable stage IIIA (T1aN2M0) right upper lobe (RUL) lung adenocarcinoma treated with chemoradiation. He remained asymptomatic for three years at which time surveillance chest CT showed a new left upper lobe (LUL) lesion diagnosed as stage I metachronous LUL lung adenocarcinoma and treated with CyberKnife Radiosurgery (CKRS). After two years, patient reported symptoms of fatigue, progressive weight loss, and generalized lymph node swelling. Chest CT showed a new right lower lobe (RLL) lesion with PET scan showing increased metabolic activity in draining lymph nodes and the liver. Image-guided core biopsy of the right supraclavicular lymph node was consistent with lung adenocarcinoma. PD-L1 expression was 0% by immunohistochemical (IHC) staining. He was enrolled in the trial described herein and blood samples were procured at baseline and after each cycle of the immunotherapy. The patient was started on two-week cycles of 240 mg nivolumab with 8 planned cycles.

**Figure 1:**
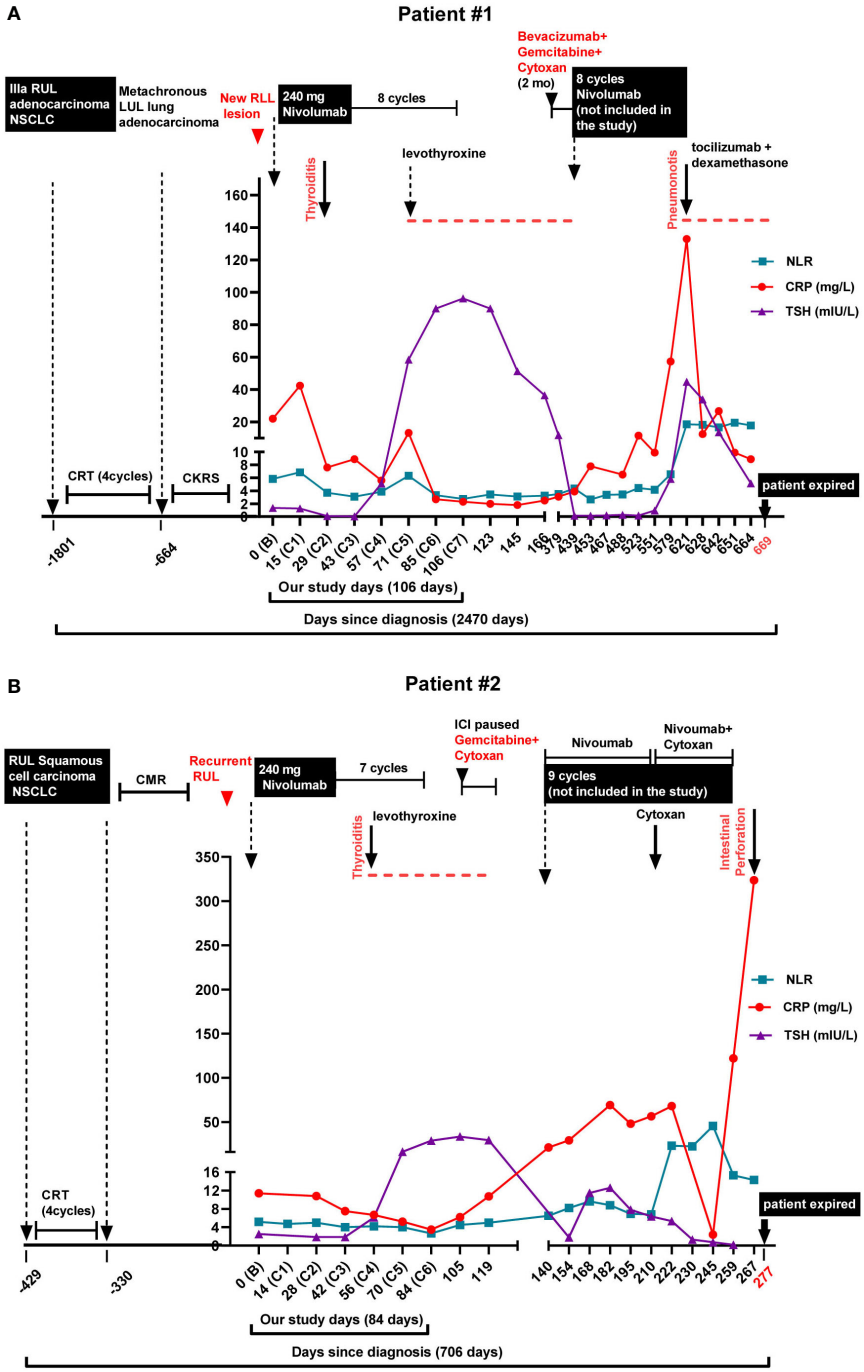
Diagnosis, treatment regimens, immune-related adverse events, and biomarker levels in the lung cancer patients. Neutrophile to Lymphocyte Ratio (NLR), C-Reactive Protein (CRP), and Thyroid Stimulating Hormone (TSH) levels are shown along with the treatment and immune-related adverse events (irAEs). **(A)** Patient #1. The patient had a 5-year history (-1801 days) of inoperable stage IIIA (T1aN2M0) right upper lobe (RUL) lung adenocarcinoma. He was initially treated with chemoradiation, followed by disease progression. He then received 8 doses of bi-weekly 240 mg Nivolumab (days 0 to 106) and was enrolled in the biomarker study. Checkpoint inhibitor-induced thyroid dysfunction (CITD, thyroiditis) was observed as an immune-related adverse event (irAE) after the 2^nd^ dose of Nivolumab (C2). Later, the patient received another round of Nivolumab treatment (days 439 to 621, not enrolled in our biomarker trial) in which pneumonitis as another irAE occurred (day 621). **(B)** Patient #2. The patient was diagnosed with RUL squamous cell carcinoma (-429 days) and received chemoradiation. Subsequent PET scan demonstrated a complete metabolic response (CMR). Unfortunately, 7 months later he had recurrence of RUL mass. The patient received 7 cycles of bi-weekly 240 mg Nivolumab treatment (days 0 to 84) when enrolled in our biomarker study. CIDT (thyroiditis) was detected after the 4^th^ dose of Nivolumab (C4). Later, the patient received another round of Nivolumab treatment (days 140 to 259, not enrolled in our biomarker trial) in which another irAE, intestinal perforation, occurred (day 267).

After receiving the second dose of Nivolumab, TSH values showed thyrotoxicosis (TSH = 0.09 and 0.04 mIU/L) at cycles 2 and 3 of Nivolumab treatment. Subclinical hypothyroidism (TSH = 5.1 mIU/L) at the 4^th^ cycle followed by symptomatic hypothyroidism (TSH = 58.37, 89.96, and 96.16) at the 5^th^, 6^th^, and 7^th^ treatment cycles, respectively was observed (**Figure 1A** and **Supplementary Figure 1A**). The patient was diagnosed with CITD and daily levothyroxine was started. The patient’s symptoms were fatigue and constipation. The dose of levothyroxine was subsequently escalated over the course of 6 months to 150 mcg daily for symptoms and thyroid function optimization. Anti-TPO antibodies were not measured.

The patient’s cancer progressed after 11 months, when PET scan showed further metastasis within the liver. The patient started on 2 months of immune modulating gemcitabine, cyclophosphamide and bevacizumab. Monthly nivolumab 480 mg cycles were restarted, but unfortunately patient was not enrolled in the trial and blood samples were not collected for T cell and cytokine analyses. The patient demonstrated an excellent response after 6 cycles of nivolumab yet developed checkpoint inhibition-induced pneumonitis (CIP). After his hospitalization for CIP, the patient began to have recurrent diarrhea and extensive workup interestingly revealed elevated levels of chromogranin and serotonin. Biopsy of the liver showed metastatic adenocarcinoma with neuroendocrine differentiation with small cell morphology, raising the concern for transformation of his primary NSCLC to SCLC. Unfortunately, a corresponding brain MRI demonstrated extensive central nervous system (CNS) disease progression and the patient expired shortly thereafter (**Figure 1A**).

### Case #2

The second patient was a 61-year-old male with history of smoking, who was initially presented with progressive neck swelling and diagnosed with superior vena cava syndrome after CT chest showed right paratracheal mass with extension into the anterior mediastinum, and SVC compression. He subsequently underwent bronchoscopy with biopsy which revealed squamous cell carcinoma and tumor cells were strongly positive for p63 and negative for TTF-1. Molecular analysis of the tumor was not performed. The patient received chemoradiation, and subsequent PET scan demonstrated CMR. Unfortunately, 7 months later the patient was admitted for post obstructive pneumonia and was found to have recurrence of RUL mass. Bronchoscopy with biopsy showed recurrent squamous cell carcinoma, with 50% expression of PD-L1 by IHC staining. He was enrolled in the trial described herein and blood samples were procured at baseline and after each cycle of the immunotherapy. The patient was started on two-week cycles of 240 mg nivolumab with 8 planned cycles.

After 4 cycles of nivolumab, routine TSH measurements revealed an increase above the upper limit of normal to 28.82 mIU/L on cycle 6, with a slight prior decrease in TSH on cycle 2 and 3 (**Figure 1B** and **Supplementary Figure 1B**). Anti-TPO antibodies were negative. The patient initially reported fatigue, and patient was started on levothyroxine 75 mcg daily. After 7 cycles of nivolumab, repeat PET scan showed progression of his disease and new liver and brain metastases. The patient was switched to gemcitabine and cytoxan, but later had hemoptysis, and nivolumab was restarted. He received an additional 9 cycles of nivolumab of which the patient was not enrolled in the trial and no blood samples were collected for our cytokine and T cell study. While on the second round of nivolumab, the patient’s dose of levothyroxine was increased to 100 mcg daily, yet TSH remained intermittently elevated ranging from 5.31 to 12.58 mIU/L while on treatment. During this time, the patient also reported constipation. Upon completing the 9^th^ cycle of nivolumab, the patient developed an intestinal perforation of undifferentiated etiology and succumbed to the complications of his disease (**Figure 1B** and **Supplementary Figure 2**).

## Discussion

Thyroid dysfunction (TD) is a common irAE that occurs in cancer patients treated with immune checkpoint inhibitors (1, 2). Subtypes and clinical manifestations of CITD are discussed elsewhere (8, 9). Multiple studies have noted that patients on ICI therapy often have a thyrotoxicosis phase prior to hypothyroidism (6, 10). Yet patients can often be asymptomatic, with one study stating that 67% of patients on ICI therapy were asymptomatic during the thyrotoxicosis phase (10). The manifestations of CITD are usually not severe, and normally grade 1 to 3 by CTCAE v5 (11). The most common clinical symptoms include fatigue, constipation, cold intolerance, swelling, and weight gain (12). Notably, both patients reported fatigue and constipation after CITD, and patient #2 had associated intestinal perforation (**Figure 1B** and **Supplementary Figure 2**). In patients with existing thyroid disease, especially with hyperthyroidism, the risk of worsening TD increases after ICI initiation (13, 14). Notably Kim et al. showed a statistically significant increase in overall survival and progression-free survival among in patients with TD when compared to euthyroid patients (15). Overall, CITD is quite common and has prognostic implications for patients’ disease. Although mild symptoms are common, severe TD is associated with significant morbidity; therefore, an increased focus on the mechanisms of CITD may improve the diagnosis, management, and care of patients.

The pathogenesis of CITD is unclear, yet the pathologic mechanisms are likely distinct from autoimmune thyroid disease, e.g., Hashimoto, subacute, and silent thyroiditis (16). While thyroid peroxidase (TPO) antibodies (Abs) are often positive in autoimmune thyroid disease, several reports have shown the TPO Abs are not necessarily associated with immunotherapy induced thyroiditis (14, 17). By contrast, Osorio et. al., showed in NSCLC receiving pembrolizumab, 80% of patients who developed CITD had TPO Abs present at diagnosis compared with 8% who did not develop CITD (6). The role of humoral immunity may have a distinct role in CITD, yet emerging data strongly implicates T cells as a primary pathologic actor (16, 18).

Other mechanistic indicators of CITD include a higher incidence with PD-1 inhibitors compared to CTLA-4 or PD-L1 inhibitors (19). Possible explanations include polymorphisms in either CTLA-4, PD-1, or PD-L1 molecules or genetic variations that predispose patients to autoimmune thyroid disease (20–22). Recent studies suggested the difference may be from the widespread T cell activation by anti-CTLA-4, whereas PD-1 or PD-L1 blockade polarizes the activation of pre-existing CD8^+^ T cells that have reactivity to thyroid antigens (16, 23, 24). The importance of T cells rather than B cells in CITD is reinforced by the reported comparison of T cells from thyroid fine needle aspiration to blood analysis showed that immunotherapy induced thyroiditis had a T lymphocyte-mediated process with intrathyroidal predominance of CD8^+^ and CD4^-^CD8^-^ T lymphocytes (18). Furthermore, Delivanis et al. suggested that ICI-related thyroid destruction may be independent of thyroid autoantibodies but involve T cell, NK cell, and monocyte-mediated pathways (14).

**Figure 2:**
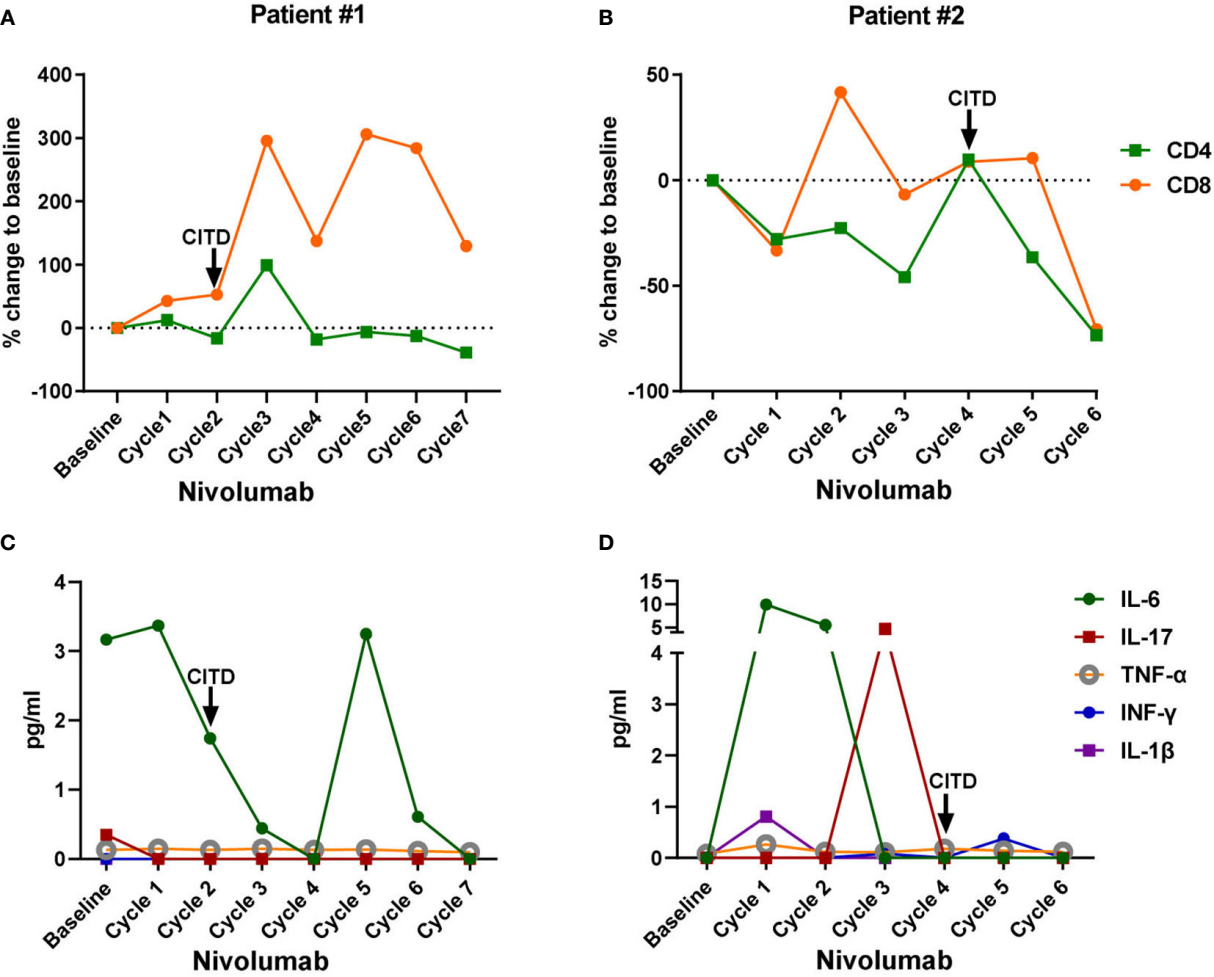
Dynamic changes of peripheral blood CD4^+^ T cells, CD8^+^ T cells, and inflammatory cytokines in the lung cancer patients treated with Nivolumab. Blood samples were collected at the baseline and after each cycle of Nivolumab treatment during the biomarker trial. Baseline: values from the blood samples collected before the Nivolumab treatment initiation; Cycle 1: values after the 1^st^ dose of Nivolumab, from the blood samples collected right before the second dose administration; Cycle 2: values after the 2^nd^ dose of Nivolumab, from the blood samples collected right before the third dose administration, and so on. **(A, B)** Percent change of peripheral CD4^+^ T cells and CD8^+^ T cells from baseline after each dose of Nivolumab in patient #1 and #2, respectively. **(C, D)** Plasma IL-6, IL-17, TNF-α, INF-γ, and IL-1β levels in patient #1 and #2, respectively. Onset of checkpoint inhibitor-induced thyroid dysfunction (CITD) is indicated by arrows on the graphs.

Interestingly, for patients described here, we observed an increase in peripheral T cells associated with the onset of CITD. For patient #1, both CD4^+^ and CD8^+^ T cells more than doubled between cycles 2 and 3 of treatment (**Figure 2A**), corresponding with a decrease in TSH to 0.09 mIU/L at cycle 2 and 0.04 mIU/L at cycle 3 (**Figure 1A** and **Supplementary Figure 1A**). For patient #2, CD8^+^ T cells more than doubled between his 1^st^ and 2^nd^ cycles of treatment (**Figure 2B**), and subsequently his TSH increased from 1.88 to 6.11 mIU/L (**Figure 1B** and **Supplementary Figure 1B**). While this observation may be in part secondary to a treatment effect as we and others have previously described, it may also go along with previous reports of T cell expansion associated with immunotherapy induced thyroiditis (25–28).

Next, we examined potential inflammatory mediators that may be associated with CITD. We were interested in measuring levels of IL-6 that we and others have found to be elevated during pneumonitis and neutropenia irAEs (25, 29). Interestingly, patient #1 had a decrease in IL-6 levels during his episode of CITD (**Figure 2C**). Patient #2 had a mildly elevated level in IL-6 to 9.97 pg/mL after the first ICI dose (**Figure 2D**), consistent with previous reports that hypothyroidism (HT) patients had elevated serum IL-6 levels (30). It is also noteworthy that patient #2 had a transient increase in IL-17 to 4.7 pg/mL (**Figure 2D**). Increased levels of IL-17 have been observed in patients with HT compared to healthy patients (31, 32). However, patient #2’s IL-6 and IL-17 levels were very low during his episode of CITD (**Figure 2D**). Furthermore, the levels of inflammatory molecules TNF-α, INF-γ, and IL-1β were very low at the onset of CITD in patients #1 and #2 (**Figures 2C, D**).

Additional proinflammatory biomarkers associated with irAEs are CRP and neutrophil to lymphocyte ratio (NLR) (33–35). We recently report a case of checkpoint inhibitor pneumonitis with increases in CRP and NLR associated with CIP onset and resolution (25). Furthermore, in patients with autoimmune hypothyroidism, a correlation was found between levels of high-sensitive CRP and thyroglobulin autoantibody and TSH levels (36). We suspected a similar observation may be consistent in CITD, yet neither patient had dynamic increases from baseline in CRP or NLR at CITD onset (**Figure 1** and **Supplementary Figure 1**). Although both patients had elevated baseline CRP levels, 22.0 and 11.4 mg/mL, respectively, after ICI treatment CRP levels decreased. However, patient #1 had a transient elevation of CRP (42.4 mg/L) after the first dose of ICI, whereas the CRP level decreased below the baseline after the second and third doses of ICI (**Figure 1A** and **Supplementary Figure 1C**). Interestingly, this patient later developed pneumonitis as an irAE while receiving Nivolumab for a second eight cycle treatment phase (not included in our biomarker trial). During this second irAE, CRP levels spiked to 132.9 mg/L, suggesting CRP as a more valuable biomarker for pneumonitis than for CITD (**Figure 1A**). Moreover, for patient#2, CRP levels continued to decrease from baseline after ICI initiation (**Figure 1B** and **Supplementary Figure 1D**). Similar to patient #1, this patient also received a second phase of Nivolumab (not included in our trial) and also developed a second irAE, intestinal perforation. On the day the patient presented with intestinal perforation, a spike in CRP level of 323.7 mg/L was observed, in contrast to the decreased CRP levels previously observed with CITD (**Figure 1B** and **Supplementary Figure 1D**). Likewise, NLR decreased during the CITD episodes after ICI treatment for both patients; however, later on during both of their second reported irAE, NLR levels elevated (**Figures 1A, B**). CRP and NLR are often appreciated as sensitive biomarkers for irAEs which are consistent with our results of the second irAEs in both patients (pneumonitis and intestinal perforation, respectively), yet these cases suggest further investigation is needed to define the utility of CRP and NLR in diagnosing and understanding CITD. It is still not fully understood why CRP and NLR levels in these two patients did not significantly elevate at the onset of CITD but spiked during the events of pneumonitis and intestinal perforation. Further studies are required to better understand the commonalities and differences among various irAEs.

CITD is a common irAE, and although cases are usually mild, and can be treated with thyroid hormone supplementation. The diagnosis remains clinically meaningful, and if left unmanaged can result in significant morbidity. Mechanistically, CITD is not fully understood, yet the histological predominance of T cells and lack of consistently elevated TPO antibodies suggest the overall importance a T cell-mediated process (5, 7, 16, 18). Consistently, our observations indicate an increase in peripheral CD8^+^ T cells during or preceding the onset of CITD in both patients (**Figures 2A, B**). Monitoring peripheral CD8^+^ T cells, in conjunction with TSH measurement, may be useful for the research and diagnosis of CITD. Recent studies demonstrate that a dynamic increase in peripheral CD8^+^ T cells is a valuable indicator of immune responses to ICIs in cancer patients (26, 27, 37). Further research is warranted to investigate the temporal and subtype changes of peripheral CD8^+^ T cells in order to elucidate immunotherapy-specific responses versus autoimmune responses in CITD.

## Conclusion

Our case studies demonstrate that increases in peripheral CD8^+^ T cells may correlate with onset of CITD. Unlike previous reports on other irAEs such as pneumonitis and neutropenia from our group and others (25, 38, 39), CRP levels and NLR were non-contributory in the diagnosis of CITD, yet larger cohorts are needed to confirm this observation. Despite the limitations of two case reports, these observations may aid in a more robust understanding of the mechanisms of CITD.

## Data availability statement

The original contributions presented in the study are included in the article/**Supplementary Material**. Further inquiries can be directed to the corresponding authors.

## Ethics statement

The studies involving human participants were reviewed and approved by The East Carolina University Institutional Review Board (IRB). The patients/participants provided their written informed consent to participate in this study.

## Author contributions

MAM: designed study, conducted, analyzed, and interpreted experiments, and wrote manuscript. JM: designed study, conducted, analyzed, and interpreted experiments, and wrote manuscript. ZH: gathered clinical data and assisted in assembling manuscript. AN: gathered clinical data, critical edits and revisions to manuscript. AH: assisted in assembling manuscript. DA: processed patient blood samples and performed experiments. SA: gathered clinical data, critical edits and revisions to manuscript. MM: enrolled patients, critical edits and revisions to manuscript. PW: supervised, enrolled patient, involved in the clinical care of patients, critical edits and revisions to manuscript. LY: supervised, designed study, analyzed and interpreted data, and wrote manuscript. All authors contributed to the article and approved the submitted version.

## Supplementary material

Supplementary Figure 1 Levels of thyroid stimulating hormone (TSH), neutrophil to lymphocyte ratio (NLR), and C-reactive protein (CRP) in the lung cancer patients treated with Nivolumab. Blood samples were collected at the baseline and after each cycle of Nivolumab treatment during the biomarker trial. **(A&B)** TSH levels (mIU/L), **(C&D)** CRP levels (mg/L), and **(E&F)** NLR levels, in patient #1 and #2, respectively. Onset of checkpoint inhibitor-induced thyroid dysfunction (CITD) is indicated by arrows on the graphs.

Supplementary Figure 2 Radiographic imaging of patient #2 during the event of intestinal perforation. **(A)** The anteroposterior chest X-ray image shows free air under the diaphragm and elevation of hemidiaphragm on the right side. **(B)** The lateral chest X-ray image shows free air below the diaphragm (red arrowhead).
