# Supplementary figures and images for: Case Report: Peripheral blood T cells and inflammatory molecules in lung cancer patients with immune checkpoint inhibitor-induced thyroid dysfunction: Case studies and literature review
Source: Front Oncol. 2022 Dec 7;12:1023545. doi: 10.3389/fonc.2022.1023545 (PMC9768626; doi:10.3389/fonc.2022.1023545)

## Supplementary Figure 1

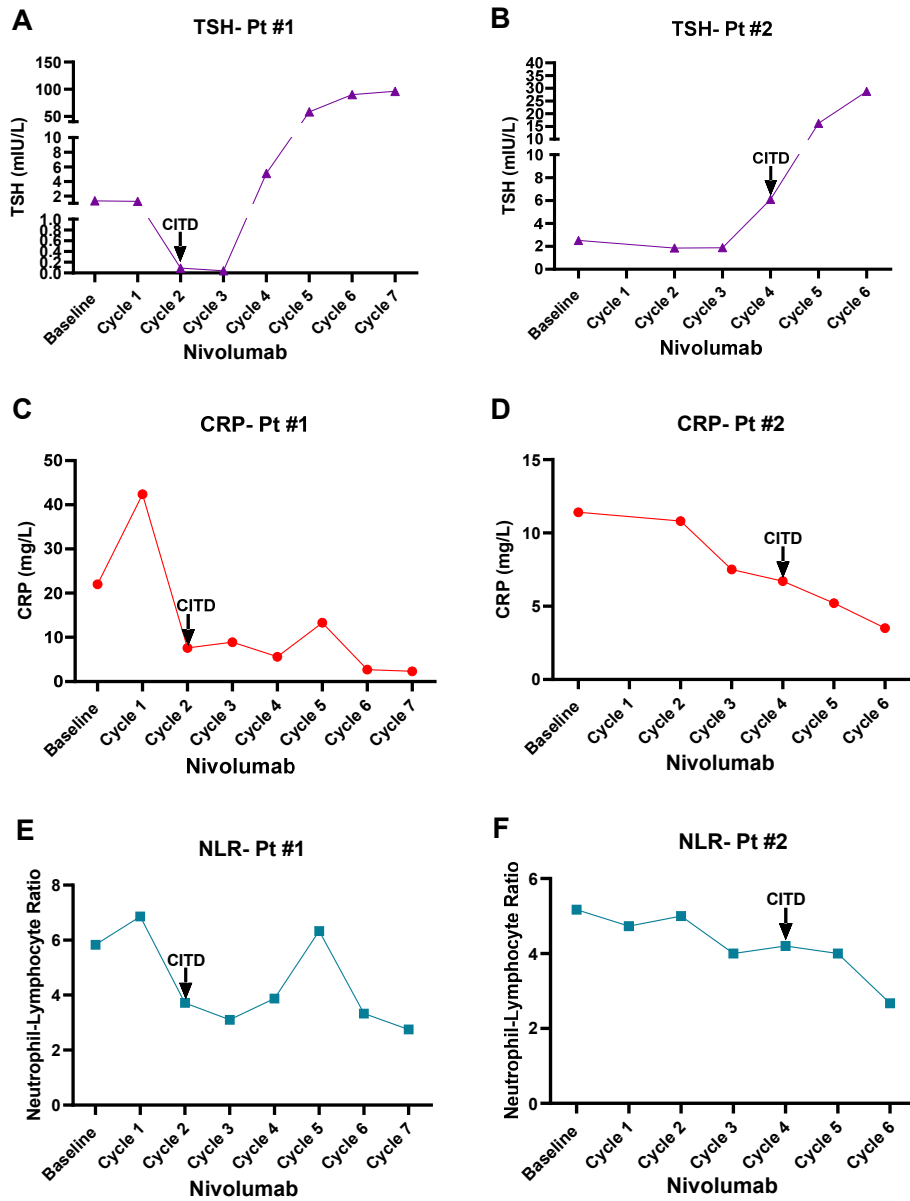

## Supplementary Figure 2

**A**

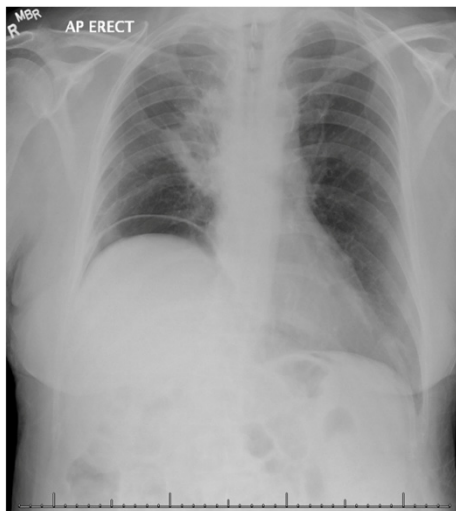

**B**

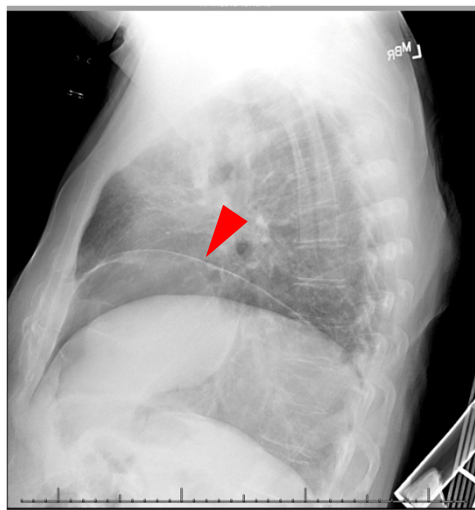

Supplement: Supplementary Figure 1 — Levels of thyroid stimulating hormone (TSH), neutrophil to lymphocyte ratio (NLR), and C-reactive protein (CRP) in the lung cancer patients treated with Nivolumab. Blood samples were collected at the baseline and after each cycle of Nivolumab treatment during the biomarker trial. (A&B) TSH levels (mIU/L), (C&D) CRP levels (mg/L), and (E&F) NLR levels, in patient #1 and #2, respectively. Onset of checkpoint inhibitor-induced thyroid dysfunction (CITD) is indicated by arrows on the graphs. [file DataSheet_1.pdf]
