# Supplementary material for: Case Report: Peripheral blood T cells and inflammatory molecules in lung cancer patients with immune checkpoint inhibitor-induced thyroid dysfunction: Case studies and literature review
Source: Front Oncol. 2022 Dec 7;12:1023545. doi: 10.3389/fonc.2022.1023545 (PMC9768626; doi:10.3389/fonc.2022.1023545)
Supplement: Supplementary file 2 [file DataSheet_2.docx]

**Materials and methods:**

**Study protocol**

The study protocol (UMCIRB 16-000719) was approved by the Institutional Review Board (IRB) at East Carolina University. Two NSCLC patients treated with PD-1 inhibitor (Nivolumab) were identified with the development of hypothyroidism and other irAEs. Patients were seen regularly at East Carolina University/Vidant Medical Center in Greenville, North Carolina.

**Blood collection, sample processing, and cytokine measurements**

Whole blood samples were collected in EDTA tubes at baseline prior to Nivolumab initiation and after each cycle of the immunotherapy. Plasma samples and CD4^+^ and CD8^+^ T cells were isolated as previously described (1). Human IL-6 Quantikine ELISA Kit (R&D Systems, D6050), human IL-17A ELISA Kit (Invitrogen, BMS2017), human TNF-α Quantikine HS ELISA Kit (R&D Systems, HSTA00E), human IFN-γ Quantikine HS ELISA Kit (R&D Systems, HSDIF0), and human IL-1β Quantikine HS ELISA Kit (R&D Systems, HSLB00D) were used to detect cytokines in plasma. ELISAs were performed in duplicates for each sample.

**Clinical and radiologic data**

Clinical and radiologic data were reviewed by two board-certified physicians. Endocrine pathologies were determined secondary to ICI treatment based on previously normal values of TSH and the clinical correlation with ICI initiation. In describing the grade of both irAEs, Common Terminology Criteria of Adverse Events (CTCAE) version 5.0 was used. Cancer stage was described according to eighth edition of the American Joint Commission on Cancer TNM staging (2).

**References**

1. McCallen JD, Naqash AR, Marie MA, Atwell DC, Muzaffar M, Sharma N, et al. Peripheral blood interleukin 6, interleukin 10, and T lymphocyte levels are associated with checkpoint inhibitor induced pneumonitis: a case report. Acta Oncologica. 2021;60(6):813-7.

2. Lababede O, Meziane MA. The Eighth Edition of TNM Staging of Lung Cancer: Reference Chart and Diagrams. The oncologist. 2018;23(7):844-8.
